# ADAR2 Protein Is Associated with Overall Survival in GBM Patients and Its Decrease Triggers the Anchorage-Independent Cell Growth Signature

**DOI:** 10.3390/biom12081142

**Published:** 2022-08-19

**Authors:** Valeriana Cesarini, Domenico Alessandro Silvestris, Federica Galeano, Valentina Tassinari, Maurizio Martini, Franco Locatelli, Angela Gallo

**Affiliations:** 1 Department of Onco-Haematology, and Cell and Gene Therapy, Bambino Gesù Children Hospital, IRCCS, 00146 Rome, Italy; 2 Department of Biomedical Sciences, Institute of Translational Pharmacology, National Research Council of Italy (CNR), 00133 Rome, Italy; 3 Department of Molecular Medicine, Sapienza University of Rome, 00161 Rome, Italy; 4 Pathology of the Adult and Developmental Age “Gaetano Barresi”, Università di Messina, 98125 Messina, Italy; 5 Department of Life Sciences and Public Health, Policlinico “A. Gemelli”, IRCCS, Catholic University of Sacred Heart, 00168 Rome, Italy; 6 Department of Gynecology/Obstetrics and Pediatrics, Sapienza University of Rome, 00161 Rome, Italy

**Keywords:** ADAR2, cancer, RNA editing, anchorage-independent growth, PTPX3, ADAM12

## Abstract

Background: Epitranscriptomic mechanisms, such as A-to-I RNA editing mediated by ADAR deaminases, contribute to cancer heterogeneity and patients’ stratification. ADAR enzymes can change the sequence, structure, and expression of several RNAs, affecting cancer cell behavior. In glioblastoma, an overall decrease in ADAR2 RNA level/activity has been reported. However, no data on ADAR2 protein levels in GBM patient tissues are available; and most data are based on ADARs overexpression experiments. Methods: We performed IHC analysis on GBM tissues and correlated ADAR2 levels and patients’ overall survival. We silenced ADAR2 in GBM cells, studied cell behavior, and performed a gene expression/editing analysis. Results: GBM tissues do not all show a low/no ADAR2 level, as expected by previous studies. Although, different amounts of ADAR2 protein were observed in different patients, with a low level correlating with a poor patient outcome. Indeed, reducing the endogenous ADAR2 protein in GBM cells promotes cell proliferation and migration and changes the cell’s program to an anchorage-independent growth mode. In addition, deep-seq data and bioinformatics analysis indicated multiple RNAs are differently expressed/edited upon siADAR2. Conclusion: ADAR2 protein is an important deaminase in GBM and its amount correlates with patient prognosis.

## 1. Introduction

Glioblastoma (GBM) is a common malignant highly aggressive brain tumor in humans affecting both children and adults that accounts for about 80% of malignant gliomas [1]. Patients diagnosed with GBM have a poor prognosis with an average survival time of 12–18 months after diagnosis [2].

A-to-I RNA editing is one of the most common epitranscriptomic events in humans and is emerging as an important player in cancer, acting as one of the primary sources of tumor heterogeneity [3,4,5,6]. A-to-I RNA editing changes nucleotides within double-stranded RNAs (dsRNAs) thanks to the action of ADAR (Adenosine DeAminases acting on RNA) enzymes. This epitranscriptomic modification can modulate both the sequence and level of RNAs by changing the splicing pattern, codons, RNA folding, and microRNA levels [7].

There are three ADAR proteins in mammals, ADAR1 (also known as ADAR), ADAR2 (also known as ADARB1), and ADAR3 (also known as ADARB2), that share a similar protein structure [8,9,10]. 

In mammals, ADAR2 is crucial for the generation of different protein isoforms starting from the same mRNA in the central nervous system [11,12], and accumulating studies are now underlining the importance of ADAR2-mediated editing in key RNAs (coding and non-coding) involved in several diseases affecting the brain [13,14,15], with its role now emerging in different cancers [16,17,18,19,20,21,22]. 

More than a thousand recoding sites were identified in humans [23,24], particularly in neuronal tissues. Recently, a weighted average over all known recoding sites, known as the recoding editing index (REI), has been introduced that well correlates with the *ADAR2* RNA level [5,25,26].

Several ADAR2 substrates have been identified to be central in GBM, acting over cell cycle checkpoints [17,19] or modulating cell migration/invasion behaviour [5,27]. Nevertheless, we are still far from having a complete picture of the role played by ADAR2 in GBM. Herein, we added a new piece of the puzzle by analyzing the distribution and expression of ADAR2 protein in tissues of GBM patients and exploring the role played by the endogenous ADAR2 deaminase in GBM cells.

## 2. Results

### 2.1. The ADAR2 Protein Level Correlates with GBM Patients’ Outcome

Previous studies demonstrated that the *ADAR2* mRNA level is extremely low compared with normal brain or astrocytes [5,28]; however, poor information concerning ADAR2 protein in GBM tissues is available. Moreover, our recent study demonstrated consistent differences between RNA and protein levels regarding the other active deaminase ADAR1 [29]. 

**Figure 1:**
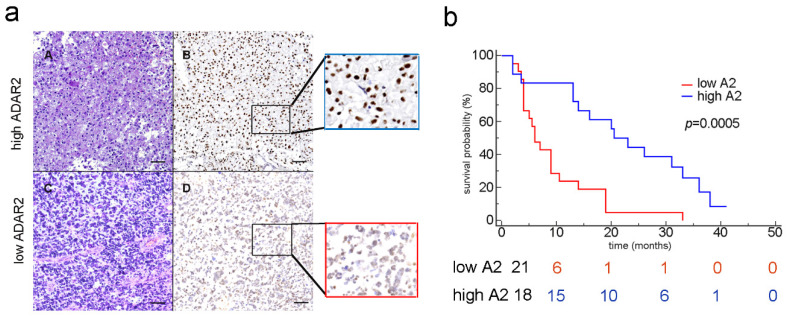
ADAR2 correlates with patients’ overall survival. (**a**) IHC analysis (H&E and the corresponding ADAR2 antibody staining) of two glioblastoma tissues (examples of high and low ADAR2 levels). Subpanels A and B show a representative GBM case with high ADAR2 expression; in subpanels C and D, a GBM case with low ADAR2 expression is shown; scale bar: 200 µm. (**b**) Kaplan–Meier curve comparing the survival of GBM patients (n = 39) stratified by ADAR2 levels. The red and blue lines represent low and high ADAR2 expression, respectively, following the scores indicated in Material and Methods (*p* = 0.0005; HR 3.954; 95% CI from 1834 to 8525).

We then analyzed the ADAR2 protein level and localization in GBM tissues (39 samples) from a cohort of homogeneously treated patients (Supplementary Table S1) by performing a series of immunohistochemistry (IHC) experiments. As a result, we found that ADAR2 protein is differently expressed among patients, with several GBM tissues (21 samples) showing low or no ADAR2 while others (18 samples) displayed a high protein level (Figure 1a). Of note, ADAR2 protein, when expressed, correctly localizes within the cell nuclei of the tumor tissues. 

Considering patient survival curves and ADAR2 protein (Kaplan–Meier analysis), we found that patients showing a low level of ADAR2 (score between 0 and 3, see Materials) exhibited a poor outcome (*p* = 0.0005) (Figure 1b, red line). The risk table indeed confirmed that, within ten months post-diagnosis, those patients showing low ADAR2 survived less (6 patients) than those who expressed a high level of ADAR2 (15 patients), and these differences were maintained over time (Figure 1b). However, no significant correlation was observed if the RNA of *ADAR2* was analyzed by interrogating The Cancer Genome Atlas (TCGA) for the glioblastoma patient cohort (data not shown). 

Overall, we show that ADAR2 protein is heterogeneously expressed among patients’ tissue samples and, when present, is correctly localized within the cells’ nuclei. Moreover, the ADAR2 protein level, but not its transcript, inversely correlates with the patients’ overall survival.

### 2.2. ADAR2 Loss Is Sufficient to Switch Cell Program to an Anchorage-Independent Growth Mode

**Figure 2:**
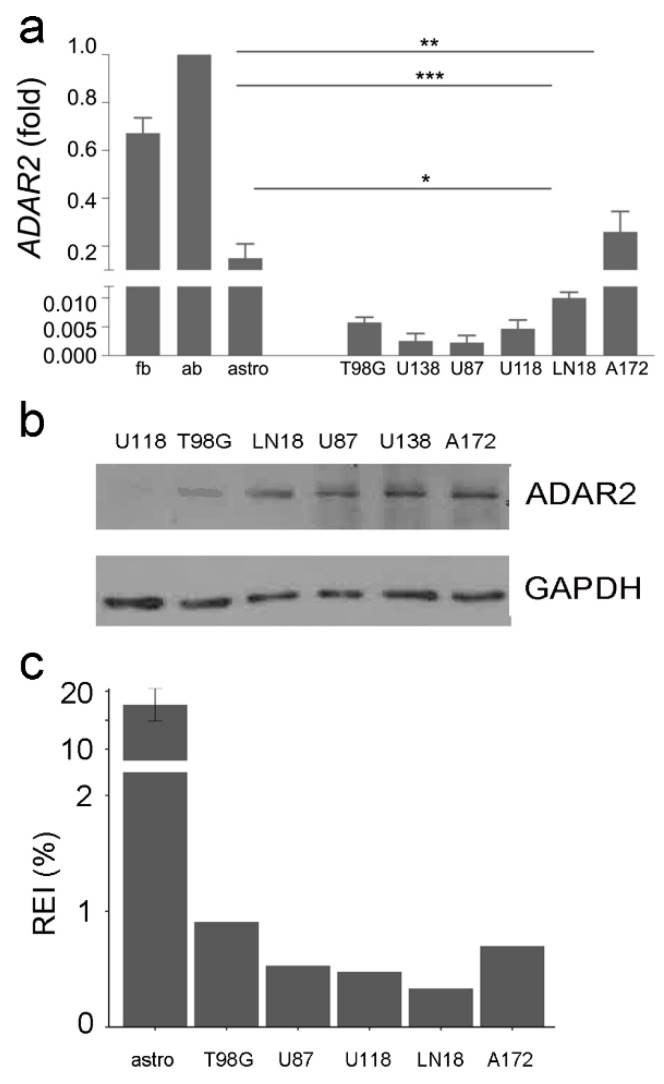
ADAR2 expression level and activity across GBM cell lines. (**a**) ADAR2 expression (qRT-PCR) in normal brain (fetal and adult), astrocytes, and glioblastoma cell lines (T98G, U138-MG, U87-MG, U118-MG, LN-18, and A172). Ct values were normalized to GAPDH mRNA levels. Mean  ±  standard deviation (*n*  =  3), values are representative as means ± SD, * *p* ≤ 0.05; ** *p* ≤ 0.01; *** *p* ≤ 0.001. Of note, ADAR2 activity is more robust in the whole brain (either adult or fetal) because of neuronal cells, where ADAR2 is highly active. (**b**) Western blotting analysis showing the ADAR2 level in glioblastoma cell lines. No control was added as the ADAR2 level was too high. (**c**) The recoding editing index (REI) in normal astrocytes (×10), T98-G, U87-MG, U118-MG, LN-18, and A172 glioblastoma cells lines is shown. REI values were calculated as the weighted average of editing levels over all known recording sites from the highly accurate list.

We analyzed the endogenous ADAR2 level (mRNA and protein) in several human GBM cell lines (A172, T98G, U118MG, U87MG, LN18, and U138-MG) that show diverse abilities to give rise to a growing tumor mass if implanted in a mouse model (www.atcc.org, accessed on 1 January 2022) (Figure 2a,b).

The ADAR2 editing activity in the cell lines was also investigated by RNA-Seq analysis, calculating the overall A-to-I editing events at the recoding sites (REI-index) (Figure 2c). Despite an overall decrease in ADAR2 expression/activity compared with controls, we found differences among GBM cell lines with discrepancies between RNA, protein and activity levels (Figure 2). The A172 cells, a non-tumorigenic cell line, display a high ADAR2 level and activity among cells (Figure 2). In contrast, the highly aggressive U87MG and U118MG cell lines show a low ADAR2 level and activity (Figure 2). 

**Figure 3:**
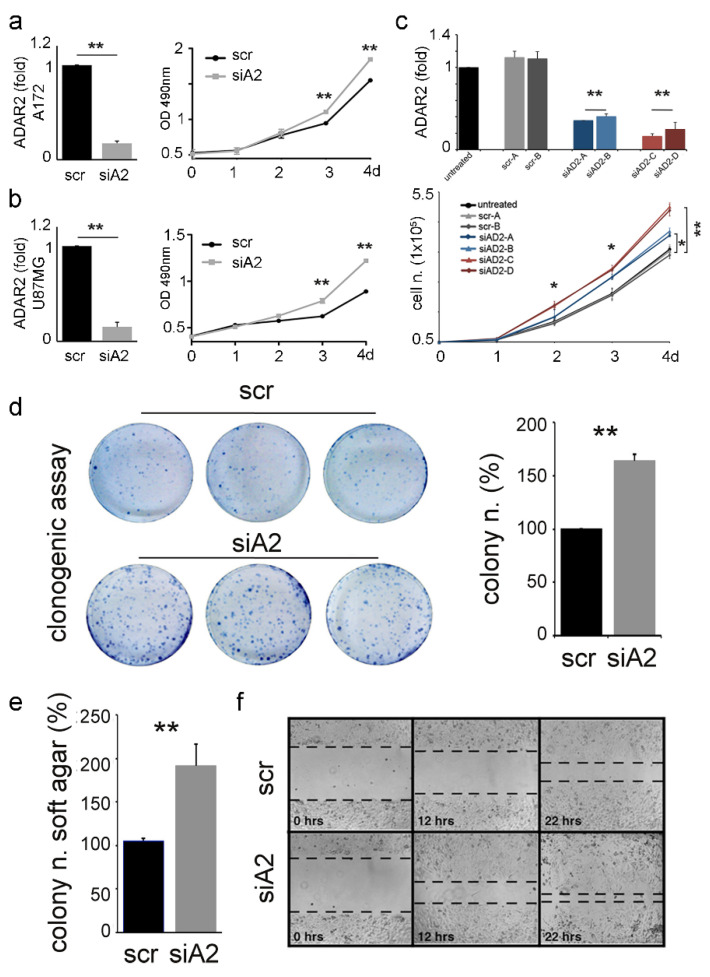
ADAR2 loss promotes proliferation, migration, and anchorage-independent growth of cancer cells. *ADAR2* knockdown (qRT-PCR at 96 h, on the left) and cell proliferation (MTS, on the right) are shown in A172 (**a**) and U87-MG (**b**) cell lines. Ct values were normalized to *GAPDH* mRNA levels. Mean ±  standard deviation (*n*  =  3), *t*-test *p*  <  0.01 **. (**c**) *ADAR2* mRNA in A172 untreated cells (black), scrambles (SCR-A, -B in light and dark grey), and different levels of ADAR2 knockdown (siAD2-A,-B in light and dark blue; siAD2-C,-D in light and dark red) are shown in A172 cells. Mean ± standard error of mean (s.e.m.) *p*  <  0.01 **. Ct values were normalized to β-actin levels. The expression levels were calculated as a relative-fold increase compared with the untreated cells arbitrarily set to 1. Below, 5 × 10^4^ of A172 un-transfected (untreated), A172 scramble (SCR-A and SCR-B), and silenced ADAR2 cells (siAD2-A, -B, -C, -D) were seeded and proliferation was monitored over 4 days. Mean ± s.e.m (n = 3), *t*-test, *p* < 0.05 *, *p* < 0.01 **. (**d**) Colony formation ability of A172 scramble (scr) and siADAR2 (siAD2) cells 10 days post-seeding (left panel, a photograph of three representative plates per cell line) quantification is shown on the right, mean  ±  standard deviation (*n*  =  4), *t*-test, *p*  <  0.01 **. (**e**) siADAR2 and control cells were seeded (2 × 10^5^ cells/well) to monitor the formation of anchorage-independent colonies after 15 days. Mean  ±  standard deviation (*n*  =  3), *t*-test *p*  <  0.01 **. (**f**) Representative photographs of scramble (scr) and siADAR2 (siAD2) A172 cells at 0, 12, and 22 hours after scratching the surface of a monolayer of cells. The wound-healing assay was performed within an interval time in which the cells do not divide.

To investigate whether ADAR2 levels may reflect distinctive cancer cell profiles, we evaluated cell proliferation fluctuations upon ADAR2 silencing in representative cell lines. First, we silenced at a similar extent (fold) the endogenous ADAR2 in A172 cells (ATCC, CRL-1620) and U87MG (ATCC, HTB-14). Decreased levels of ADAR2 enhanced cell proliferation in both cell lines (Figure 3a,b, Supplementary Figure S1a). Then, we generated stable clones of siADAR2 in A172 cells (an attempt that failed in all the other cell lines), and clones expressing ADAR2 at different levels were selected. We found that an ADAR2 progressive decrease leads to a gradual increase in cell proliferation, with the minimal ADAR2 level corresponding to the more aggressive cell behavior in terms of cell proliferation (Figure 3c, Supplementary Figure S1b). 

Stable siADAR2-A172 (with >50% decreased ADAR2 level, Supplementary Figure S1c) and control were generated and tested for their ability to grow in an anchorage-dependent and -independent mode. We report that a significantly higher number of anchorage-dependent colonies were generated by siADAR2 cells (Figure 3d), and this is independent of the cell number seeded (1000 or 1500 cells/well) (data not shown). Then, we tested whether the silenced cells also acquired the ability to grow independently of a solid surface. To this aim, we performed a soft-agar colony-formation assay, and cells were seeded and left to grow for two weeks. The assay indicated a significantly higher number of colonies in the siADAR2 cells (doubling their number) than the control (Figure 3e). Finally, we found that siADAR2 cells showed higher migratory behavior than control cells (Figure 3f).

Overall, our data indicate that ADAR2 loss switches cell programs towards a more aggressive cell behavior and an anchorage-independent growth mode.

### 2.3. Modulation of Cancer-Related Genes upon ADAR2 Silencing

**Figure 4:**
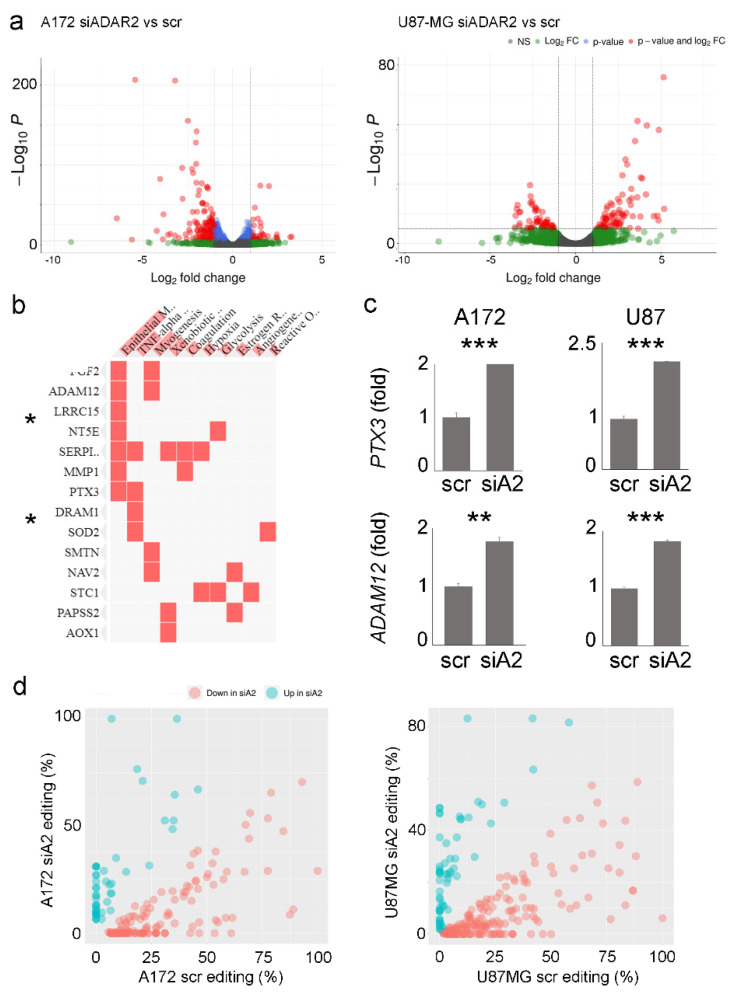
Gene expression profile and RNA editing pattern changes upon ADAR2 silencing. (**a**) Volcano plots showing the effect of ADAR2 silencing on the A172 and U87-MG cell lines transcriptome in terms of significance levels and fold changes in gene expression as calculated by DESeq2. The cut-off for log2FC is >|2| and the cut-off for the p.adj value is 1 × 10^−6^. (**b**) Gene enrichment analysis showing the pathways in which common genes are significantly upregulated in A172 siADAR2 and U87MG siADAR2, * *p* < 0.05. (**c**) qRT-PCR showing the expression levels of PTX3 and ADAM12 transcripts in control and siADAR2 A172 and U87-MG cells. Mean ± standard deviation *t*-test, ** *p* ≤ 0.01, *** ≤ 0.001. (**d**) Scatter plots reporting the editing levels (%) for sites significantly (*t*-test, *p*-value < 0.05) modulated between scramble (scr) versus silenced ADAR2 (siA2) cell lines. The under-edited sites in siADAR2 are shown in light red, while the upregulated sites are shown in cyan.

To identify genes being modulated/edited by ADAR2 in siADAR2 GBM cell lines, we performed deep-sequencing analysis of RNA extracted from controls and siADAR2 A172 and U87MG cells. Three replicates were sequenced for each condition in both cell lines, from which some “outliers” were discarded on the basis of the sample-to-sample distance matrix (Supplementary Figure S2a). Bioinformatics analysis of RNA-Seq data highlighted the presence of 1339 and 987 transcripts significantly modulated (FDR ≤ 0.05) in both cell lines, respectively (Figure 4a and Supplementary Table S2). We concentrated on genes significantly overexpressed in both cell lines following ADAR2 silencing. Using gene list enrichment analysis tools (i.e., Enrichr [30]), we found some shared molecular pathways belonging to specific cancer signatures, such as hypoxia, glycolysis, and epithelial–mesenchymal transition pathways, with the latter being the most representative one, with seven genes (Figure 4b). Interestingly, among the seven genes upregulated by ADAR2 in both of the GBM cell lines appeared ADAM12, MMP1, and SERPINE1, which were recently identified as key extracellular matrix (ECM) regulator genes in several cancer types [31]. Moreover, ADAM12 was previously identified among the ADAR2-edi-miR-589-3p-modulated genes [16]. Together with ADAM12, the glycoprotein pentraxin-3 (PTX3) is also a critical protein promoting cell invasiveness of various cancers [32]. Therefore, we validated by qRT-PCRs the expression of both ADAM12 and PTX3 in siADAR2 glioblastoma cells (A172 and U87MG) (Figure 4c).

A genome-wide screening of the RNA editing profile in both A172 and U87MG cell lines (siADAR2 and controls) was also performed. Taking advantage of the strand-oriented feature of the analyzed RNA-Seqs, de novo research of the edited sites was performed with an (as expected) enrichment of A/G mismatches to indicate a good signal-to-noise ratio (Supplementary Figure S2b). As a result, we identified 218 and 243 differentially edited sites in siADAR2 and controls in A172 and U87MG cell lines, respectively (*t*-test, *p*-value < 0.05), with most of the sites showing a significant decrease in editing (Figure 4d and Supplementary Table S3). Almost all of the modulated sites are localized within Alu repeats with a preferential enrichment in the 3’UTR according to the Gencode v40 annotation. 

## 3. Discussion

Glioblastoma is a fast-growing malignant brain tumor still incurable in both children and adults. Genetic and epigenetic alterations are key mechanisms in GBM development and dictate the criteria for cancer classification by altering both sequences and expression of target transcripts [33,34,35]. Additionally, recent studies underlined the essential role of epitranscriptomic mechanisms in cancer, such as A-to-I RNA editing mediated by the active ADAR enzymes (ADAR1–2) [36]. By introducing Inosines within the target genes, editing enzymes alter the sequence, splicing, folding, transport, binding, and expression of multiple RNAs. Therefore, A-to-I RNA editing events can contribute to cancer heterogeneity and progression [3,5]. ADAR2 RNA level and editing activity are decreased in GBM compared with controls [5,17,18,28,37], and several ADAR2 target genes have been identified as critical players in GBM proliferation/migration, indicating ADAR2 editing to be an important event to inhibit GBM progression [5,16,27]. On the contrary, the ADAR1 protein upregulation, promoted by METTL3, plays a protumoral role in GBM, yet in an editing-independent mode [29]. 

Previous studies on RNA editing and cancers are mainly based on the whole transcriptome analysis of homogenized tissues (including different cell types) and reported as the average of multiple patients’ tissues; however, this approach can mask differences among different patients and among diverse tissues/cells and does not take into consideration the ADAR protein level.

Indeed, there is a lack of studies on the ADAR2 protein expression pattern in GBM tissues, in the light of recent research underlining discrepancies between the RNA/protein of the other active human deaminase ADAR1 [29] and in Drosophila dADAR [38].

Herein, we performed IHC on GBM samples and found that ADAR2 protein is heterogeneously expressed among different patients, with some cancer tissues showing low or no ADAR2, and others presenting a high protein level that is correctly localized within cell nuclei. This finding is quite surprising as a substantial overall decrease in *ADAR2* RNA has been previously reported. Moreover, we show that the ADAR2 protein level, but not its RNA, inversely correlates with patients’ overall survival, identifying ADAR2 protein as a possible prognostic factor. 

Discrepancies between protein and RNA of ADAR2 and GBM patients’ survival could be explained by taking into account different factors: (1) the expression of the ADAR2 protein in the nucleus was analyzed only in the cancer cells, while TCGA evaluates the overall expression of *ADAR2* RNA in the tumor mass; (2) the patients we analyzed are part of a unicentric cohort of GBM patients with a homogeneous clinical-therapeutic treatment, differently from the TCGA cohort; and (3) post-transcriptional modification events can introduce differences between the ADAR2 transcript and its protein, as recently shown with ADAR1 [29]. Yet, future studies on a larger cohort of GBM patients will be necessary to further investigate what is reported herein.

Together with GBM tissues, different patterns of ADAR2 protein levels were also detected among GBM cell lines. Decreasing the endogenous ADAR2 levels is sufficient to accelerate cell proliferation regardless of the original ADAR2 protein level. Moreover, cells showing non-tumorigenic ability *in vivo* and high ADAR2 protein levels can switch from an anchorage-dependent to an anchorage-independent growth mode by decreasing the ADAR2 level. 

Deep-sequencing analysis of scr and siADAR2 in A172 and U87MG cell lines highlighted the presence of genes modulated in both cell lines and belonging to the hypoxia, glycolysis, and epithelial–mesenchymal transition pathways, being significantly upregulated upon ADAR2 silencing. Both PTX3 and ADAM12 are among the upregulated genes in siADAR2 cells (in both A172 and U87MG cell lines), and both of these genes belong to the epithelial–mesenchymal transition pathways. The overexpression of ADAM12 upon ADAR2 silencing fitted well with previous results indicating ADAM12 as an indirect ADAR2 target gene [16]. Indeed, ADAR2 edits at an extremely high level (almost 100%) the miR-589-3p within its *seed* sequence in the normal brain, while a consistent editing decrease at this site is observed in glioblastoma. The edited version of miR-589-3p targets and inhibits ADAM12, while the loss of editing releases ADAM12 transcript, which can be expressed, increasing cell invasion and proliferation. We analyzed miRNome from scr and siADAR2 cell line (A172) and found a significant reduction in editing events within the miR 589-3p *seed* (data not shown). 

We reported that ADAR2 can control the level of the glycoprotein pentraxin-3 (PTX3). PTX3 is a promoter of cell invasiveness and is involved in the progression of various cancers, including glioblastoma, where it correlates with the disease grade [39], thanks to its downstream target genes involved in migration and invasion [40]. This is the first study showing an epitranscriptomic control of PTX3 that can be of great interest for a better understanding of the role/action of PTX3 in different cancers and for future therapeutic interventions crossing immunity, tissues remodeling, and cancer [41]. Indeed, epitranscriptomic changes, which include multiple RNA modification events, is emerging as an important player in cancer [42].

GBM is characterized by highly invasive and infiltrative cells, leading to tumor recurrence and poor prognosis. Herein, we demonstrated that ADAR2 could be a possible prognostic factor and a potential therapeutic target as it inhibits key cancer features and controls multiple essential genes important to GBM progression. 

## 4. Materials and Methods

### 4.1. Human Tissues

This study includes a unique collecting center including a cohort of 39 unselected adult patients, collected from 2018 to 2019, with GBM in the supratentorial compartment. Patients underwent craniotomy for tumor resection at the Institute of Neurosurgery (Policlinico “A. Gemelli”, IRCCS, Catholic University of Sacred Heart, Rome, Italy) with the histology confirmed GBM (World Health Organization grade IV; Università Cattolica del Sacro Cuore di Roma, UCSC). Immunophenotypical characterization, as well as molecular analysis of O6-methylguanine-DNA methyltransferase (MGMT) promoter methylation and isocitrate dehydrogenase 1 or 2 (IDH1/IDH2) mutation, were assessed as previously described [43]. The patients were treated postoperatively homogeneously with adjuvant radiotherapy and temozolomide (TMZ, Stupp protocol). Patients, aged from 42 to 72 years at the time of primary surgery, received radiotherapy and concomitant TMZ followed by six cycles of adjuvant TMZ according to the Stupp protocol [43]. The main clinical and biological features of the GBM patients are reported in Supplementary Table S1. All patient data were collected anonymously; written informed consent, as part of the routine diagnosis and treatment procedures, was obtained from patients or their guardians according to the Declaration of Helsinki; and the study adhered to good clinical practice guidelines. Overall survival (OS) was calculated from the date of surgery when a diagnosis of GBM was established until death.

### 4.2. Cell Culture and ADAR2 Knockdown

The well-characterized human glioblastoma cell lines T98G, U138MG, U87MG, U118MG, LN18, and A172 were obtained from American Type Culture Collection (ATCC) and routinely maintained in Dulbecco’s modified Eagle’s medium (DMEM) supplemented with 10% fetal bovine serum (Gibco-Life Technologies, Carlsbad, CA, USA), 100 U/mL penicillin, and 100 μg/mL streptomycin at 37 °C in 5% CO_2_. All of the cell lines originated from adult male patients. Human primary astrocytes (NHA–Human Astrocytes, CC-2565, Lonza Group AG, Basel, Swiss) were maintained for a few passages in ABM^TM^ astrocyte cell basal medium following the manufacturer’s instructions. Both fetal (cat n. 636530) and adult (cat n. 636526) human brain RNA were obtained from Clontech-Takara, Kyoto, Japan. For *ADAR2* transient silencing in A172 and U87MG cells, the *ADAR2* siRNA pool (ON-TARGETplus SMARTpool for the siRNA, 1: GCCCAGGACUCAAGUAUGA, 2: GCAAUGGCCACUCCAAGUA, 3: GGAGAUCCUUGCUCAGAUU, and 4: ACAUGAACUGAACGGUUAA) and the negative control pool (ON-TARGET Plus Nontargeting pool, 1: UAGCGACUAAACACAUCAA, 2: UAAGGCUAUGAAGAGAUAC, 3: AUGUAUUGGCCUGUAUUAG, and 4: AUGAACGUGAAUUGCUCAA) were used in accordance with the manufacturer’s instructions (Dharmacon). The BLOCK-iT^TM^ inducible Pol II miR RNAi Expression Vector Kit by EmGFP (Invitrogen, Waltham, MA, USA) was used to obtain stable A172 cell lines silenced for *ADAR2* and its control. The specific sequence (UAAUCUUGGAGCGUAGUAAGU) to silence *ADAR2* (selected by Block-iT RNAi Designer) and the scramble sequence (provided by the kit) were cloned in the pcDNA^TM^6.2-GW/EmGFP-miR vector according to the manufacturer’s instructions. A172 cells were transfected with Lipofectamine 2000 (Invitrogen-Life Technologies). For stable clones, the cells were maintained under blasticidin selection (20 μg/mL). Immediately after transfection, cells were selected according to their GFP expression units (MIF = mean index fluorescent). 

### 4.3. RNA Isolation

Total RNA was isolated using TRIzol reagent (Invitrogen, Waltham, MA, USA). The procedure was performed according to the manufacturer’s recommendations. RNA concentration and purity (A260/A280 nm ratio) were evaluated using a NanoDrop ND-2000 (Thermo Scientific, Waltham, MA, USA). RNA quality was assessed by gel electrophoresis or by an Agilent 2100 Bioanalyzer microfluidics-based platform (Agilent Technologies, Santa Clara, CA, USA).

### 4.4. Real-Time PCR (qRT-PCR)

Here, 1 μg of total RNA (pre-treated with DNase I) was used to generate cDNA by the ImProm-II Reverse Transcription System (Promega Corporation, Madison, WI, USA) using random hexamer primers according to the manufacturer’s instructions. *GAPDH* and/or *β-Actin* were used as controls for the normalization of mRNAs. The relative amount of each substrate was calculated by the 2^−ΔΔCt^ method [44]. Expression levels were represented as a relative-fold increase compared with the control sample, arbitrarily set to 1. All qRT-PCR reactions were performed in duplicates and repeated at least two times from independent experiments. All of the primers were supplied by Applied Biosystems: *ADAR2*, ID Hs00953730_m1; *GAPDH*, ID Hs99999905_m1; *β*-actin, ID Hs99999903_m1; ADAM12, ID Hs01106101_m1; and PTX3, ID Hs00173615_m1.

### 4.5. Immunoblotting

Total protein extracts were isolated with RIPA lysis buffer in the presence of a protease inhibitor mixture and phosphatase inhibitor cocktail (Sigma-Aldrich, Burlington, MA, USA). Protein extracts were quantified with BCA Protein Assay Kit (Pierce Biotechnology, Inc., Waltham, MA, USA). Equal amounts of total cellular lysates (30 μg) were separated by SDS-PAGE, transferred on nitrocellulose membrane, analysed by immunoblotting with the appropriate antibodies, and then revealed by ECL (GE Healthcare). The antibodies used in this study were anti-ADAR2 (1:200, Sigma-Aldrich, Burlington, MA, USA) and anti-*β*-actin (1:5000, Santa Cruz Biotechnology, Santa Cruz, CA, USA). 

### 4.6. Immunohistochemistry

Formalin-fixed, paraffin-embedded sections (3 μm thick) were mounted on positively charged glass slides. Deparaffinization and antigen retrieval were performed using the PT link instrument (Agilent Dako, Santa Clara, CA, USA) and the EnVisionTM FLEX, low pH solution (Agilent Dako, Santa Clara, CA, USA). Endogenous peroxidase was blocked by hydrogen peroxide (Sigma-Aldrich, Burlington, MA, USA), and then sections were incubated ON at 4 °C with mouse monoclonal antibody anti-ADAR2 (1.3.1): sc-73409, 1:100 dilution (Santa Cruz Biotechnology, Santa Cruz, CA, USA) followed by EnVision FLEX/HRP (Agilent Dako, Santa Clara, CA, USA). 3,3′-Diaminobenzidine was used as the enzyme substrate to observe the specific antibody localization, and Mayer hematoxylin was used as a nuclear counterstain. The staining intensity of tissue slides was evaluated independently by two observers (V.C. and M.M.) who were blinded to the patients’ characteristics and survival, as previously described [29]. Cases with disagreement were discussed using a multi-headed microscope until agreement was achieved. To assess differences in staining intensity, an immunoreactivity scoring system was applied. ADAR2 expression in each specimen was scored according to the extent (percent of stained cells) and intensity of nuclear expression staining. The score for the percentage of stained cells was scaled as 0 for no IHC signal at all, 1 for 1–30%, 2 for 31–70%, and 3 for 71–100% of tumor cells stained. The score for IHC intensity was scaled as 0 for no IHC signal, 1 for weak, 2 for moderate, and 3 for strong IHC signals. The final score used in the analysis was calculated by multiplying the extent score and intensity score, with a maximum score equal to 9. An immunohistochemical score between 0 and 3 was defined as a low protein expression level, while a score from 4 to 9 was defined as a high protein expression level.

### 4.7. Statistical Analysis

The statistical analyses were performed using two-tailed Student’s *t*-test and the values are represented as means ± SD, and statistical significance was set at *p* ≤ 0.05. Kaplan-–Meier survival curves were plotted and differences in survival between groups of patients were compared using the log-rank test and Gehan–Breslow–Wilcoxon performed by GraphPad Prism 7 software. Overall survival (OS) was calculated from the date of surgery to death or end of follow-up. Experiments were repeated independently multiple times and similar results were obtained.

### 4.8. MTS Colorimetric Assay

Here, 2 × 10^3^ cells/well were seeded onto a 96-well plate and cell proliferation was measured daily by3-(4,5-dimethylthiazol-2-yl)-5-(3-carboxymethoxyphenyl)-2-(4-sulfophenyl)-2H-tetrazolium inner salt (MTS) using CellTiter 96 AQueous One Solution Cell Proliferation Assay (Promega Corporation, Madison, WI, USA). The absorbance intensity was determined on a microplate reader at 490 nm. The assay was repeated three times in triplicate.

### 4.9. Proliferation and Clonogenic Assay

Here, 5 × 10^4^ cells were seeded in 35 mm dishes and live cells (Trypan blue dye exclusion) were determined daily, from day 1 to day 4. The assay was repeated at least three times in duplicate. To evaluate cell colony-forming ability, cells were seeded in 60 mm dishes with 1000 or 1500 cells per dish and, after 10 days, the colonies were stained with methylene blue and counted (>50 cells equaled one colony). The assay was repeated four times in triplicate.

### 4.10. Soft Agar Assay

The knocked-out *ADAR2* and control cells were mixed with DMEM (10% FCS) medium containing 0.33% Noble agar (Sigma-Aldrich, Burlington, MA, USA). Then, 2 × 10^5^ cells were re-suspended and seeded in triplicate into six-well plates coated with 0.5% agar in DMEM (10% Fetal Calf Serum). After solidification at room temperature, plates were incubated in a 5% CO_2_ incubator at 37 °C for 15 days and the medium was refreshed every 5 days. The number of colonies per field was counted under the contrast-phase Eclipse E600 microscope (Nikon Corporation, Tokyo, Japan). Two independent experiments were carried out in triplicate.

### 4.11. Monolayer Wounding Assay

For the evaluation of in vitro cell motility, a monolayer-wounding assay was performed. Cells were allowed to form a monolayer on a culture dish surface and, when they were approaching 100% cell confluence, a wound was made by scratching the monolayer with a pipette tip. After the scratching, the cells were incubated in 5% CO_2_ at 37 °C for a further 24 h. Photographs (10× magnification using Leica DMi8 microscope) of the wound were taken at various time points. Three independent series of experiments were performed. The wound area was measured by the program Image J software (NIH, Bethesda, MD, USA). The percentage of wound closure was estimated by the following equation: wound closure % = [1 − (wound area at Tt/wound area at T_0_)] × 100%, with T_0_ being the time immediately after wound and T_t_ being the time 12 h post-wound.

### 4.12. Bioinformatic Data Analysis

RNA-seq experiments from normal astrocytes from the adult temporal lobe (ten biological replicates) and glioblastoma cell lines (A172, U87MG, U118MG, LN18, and T98G) were downloaded from the NCBI SRA portal, respectively, as part of the SRP064454 dataset [45] and the Cancer Cell Line Encyclopedia (SRP186687) project, and transformed into fastq files. mRNA-sequencings for the ADAR2-silenced A172 and U87MG glioblastoma cell lines were performed with a stranded and paired-end protocol on the Illumina NextSeq 500 platform at GenomiX4Life, thus obtaining ~80 million total reads per sample (2 × 75 bp). All of the preprocessing steps to trim the adapters and remove the low-quality reads were performed with the dedicated fastp [46] tool version 0.23.2. The mean quality per base was fixed at a phred-score of 20 and reads with more than 30% of unqualified bases (-q20 -u30-l55 --detect_adapter_for_pe) were removed. Reads shorter than 55 bases were also removed. GRCh38/hg38 human reference genome was used for all analyses. Cleaned reads were aligned with STAR (2.7.9a) [47] using the ENCODE standard options onto a consensus version of the reference (GRCh38) human genome. The 1000 Genomes Project VCF file with consensus SNVs and InDels was provided at the genome generation stage and the alternative alleles in this VCF were inserted to the reference genome to create a ”transformed” genome. At the mapping stage, the reads were mapped to the transformed genome and the alignments were transformed back to the original (reference) coordinates. Alignments in BAM format were sorted by genomic coordinates and indexed by SAMtools. PCR duplicates were marked using Picard tools (MarkDuplicates) version 2.25.4 and considered only for RNA editing analysis. Gene expression analysis: quantification was obtained with the featureCounts tool of the Subread [48] 2.0.2 package (parameters ‘--countReadPairs’ and ‘-p’) and the gencode annotation v40. The counts’ normalization and differential gene expression analysis were performed using the well-known Bioconductor package in R DESeq2 [49] version 1.36.0, following the manufacturer’s instructions. Gene set enrichment analysis was performed using the Enrichr web tool [50]. 

RNA editing analysis: candidate RNA editing sites were detected using a slightly modified version of the protocol recently published by Lo Giudice and Picardi [25] based on the REDItools [51] python suite and the use of a differential filtering scheme with RNA editing candidates in repetitive non-*Alu* regions and non-repetitive regions undergoing more stringent filters compared with those applied for sites in *Alu* repetitive elements. Briefly, after the first round of the REDItoolDnaRna script and subsequent filtering (sites reported in dbSNP v.151 or cell lines specific somatic mutations obtained from CCLE were removed), reads harboring the variations were extracted and re-aligned using PBLAT [52]. The putative editing sites obtained were further subjected to a second run of REDItool excluding duplicated reads and sequences known to align on multiple genome locations by Blat. The remaining A-to-G variants were finally annotated using ANNOVAR and Gencode genes. Differential editing analysis was performed by comparing editing levels in controls and siADAR2 samples with unpaired Welch’s *t*-test, and significantly differentially edited sites were considered those that showed a *p*-value < 0.05. Plotting and statistics were carried out using specific R packages.

## 5. Conclusions

Epitranscriptomic changes play an emerging role in cancer and include several RNA modification events [42]. Among them, A-to-I RNA editing mediated by the ADAR enzymes is one of the most abundant in humans. Herein, we show that ADAR2 loss promotes cell proliferation and switches the cell program towards more aggressive cell behavior. Most importantly, we report that the ADAR2 protein level can be considered a possible prognostic factor for GBM patients as it is positively associated with patient overall survival.

## Data Availability

The data presented in this study are available on request from the corresponding author.

## Supplementary Materials

The following supporting information can be downloaded at: https://www.mdpi.com/article/10.3390/biom12081142/s1, Figure S1: western blotting analysis of siAdar2 cells lines; Figure S2: heatmap, clustering and Distributions of single nucleotide variants of de novo RNA editing analysis; Table S1: Clinical and pathological features of Glioblastoma patients; Table S2: gene expression analysis of siADAR2 cells vs controls; Table S3: RNA editing analysis of siADAR2 cells vs controls.
