# Supplementary material for: ADAR2 Protein Is Associated with Overall Survival in GBM Patients and Its Decrease Triggers the Anchorage-Independent Cell Growth Signature
Source: Biomolecules. 2022 Aug 19;12(8):1142. doi: 10.3390/biom12081142 (PMC9405742; doi:10.3390/biom12081142)
Supplement: Supplementary file 1 [file biomolecules-12-01142-s001.zip › Supplementary Figures S1-S2.pdf]

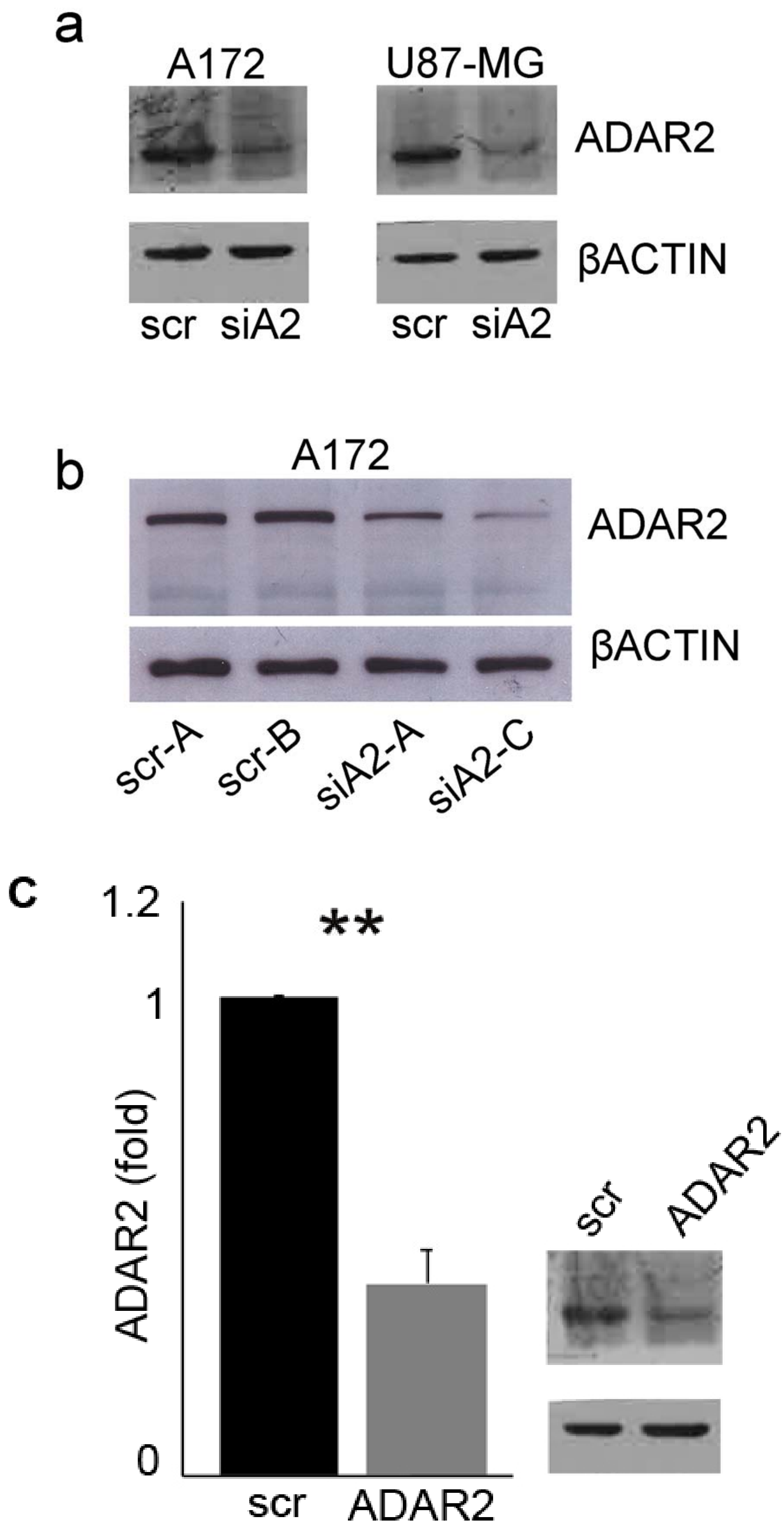

Figure S1: **(a)** Western blotting analysis of transient scramble (scr) and siADAR2 (siA2) A172 and U87-MG cell lines. **(b)** Western blotting analysis of two stable clones of scrambles (SCR-A and SCR-B), and two representative clones at different levels of ADAR2 knockdown (siA2-A and siA2-C) in A172 cell lines. (**c**) qRT-PCR and western blotting analysis of scramble (scr, black) and siADAR2 (siAD2, grey, with >50% decreased ADAR2 level) cells. Mean ± standard deviation ($n = 3$), t-test $p < 0.01$ **

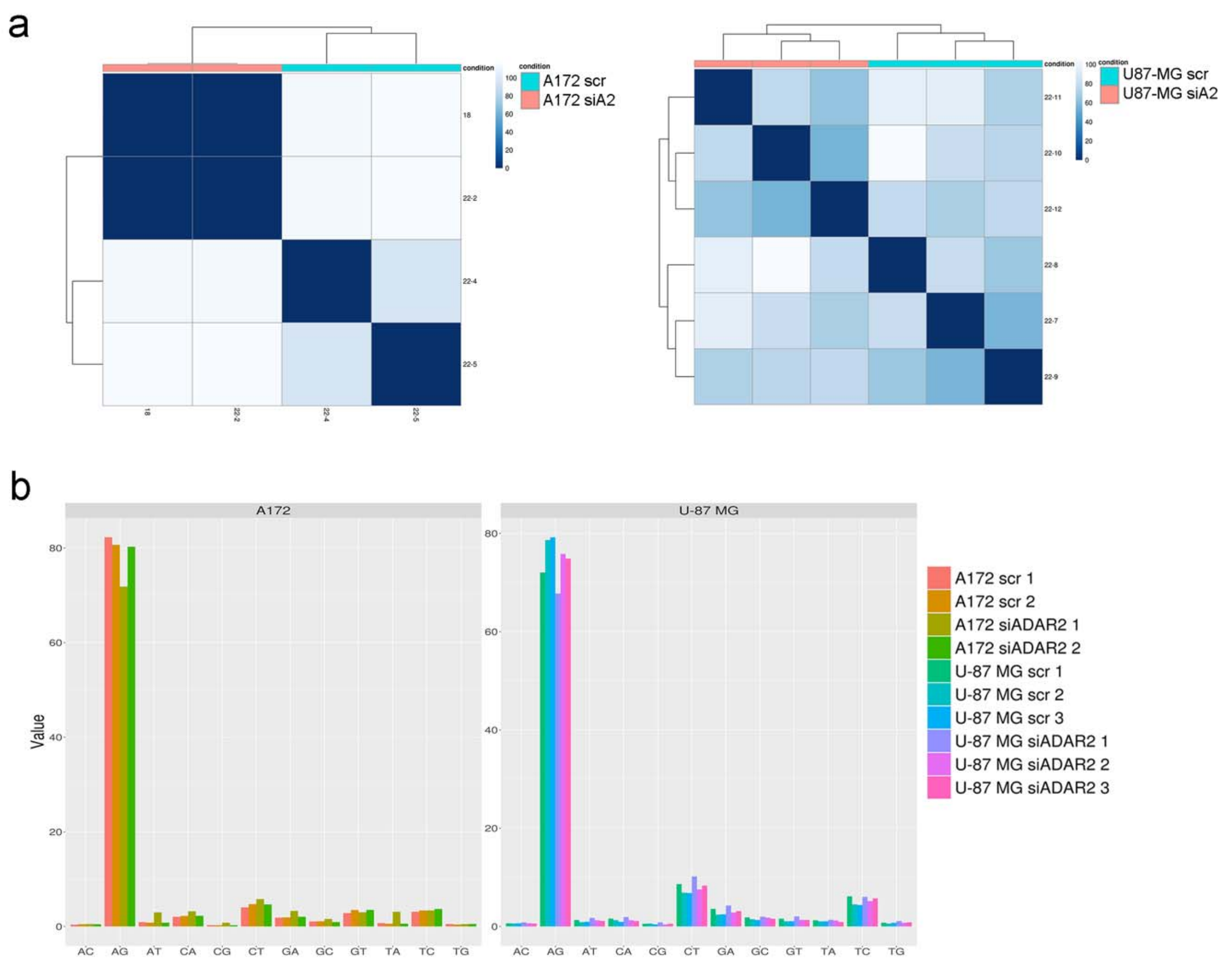

Figure S2: **(a)** Heatmap and clustering of sample-to-sample distances based on gene expression for A172 (left) and U87-MG (right) GBM cell lines. **(b)** Distributions of single nucleotide variants detected by REDItools applying the protocol for *de novo* research of RNA editing.
